# Abrasions of the Teeth

**Published:** 1897-04

**Authors:** Harry Benedick Respinger

**Affiliations:** Basle, Switzerland


### THE DENTAL REGISTER.

VOL. LI.] APRIL, 1897. [No. 4.

### COMMUNICATIONS.

### Abrasions of the Teeth.

BY HARRY BENEDICK RESPINGER, D.E., D.G., D.D.S., BASLE, SWITZERLAND.

A large number of specialists have considered the question of dental abrasion, yet it is surprising to see how few have viewed this affection histologically.

Wedl, Baume and Schlenker have undoubtedly given the best microscopical description of this lesion, and as even they were far from being precise and complete, I was prompted to make some original researches in this direction, utilizing a large number of teeth abraded in different degrees. Careful microscopical examinations permitted me to establish the following facts: The abraded surface presents a neat and distinct line which, however, is never perfectly straight. In nearly every case I was able to detect slight undulations on this surface, contrary to the statements of different authors, which can not possibly be compared with the semi-lunar lacunæ of Howship; this aspect is maintained on the surface of both the enamel and the dentin. The prisms of the enamel are neatly abraded and present no traces of disintegration in the vicinity of the abrasion. On the surface of the lesion the free extremity of the dental canaliculi is marked by a rounded section protruding like the head of a gimp-tack; the diameter of this projection is somewat greater than that. On examining the oral cavity of a normal human being we are soon able to discern that the masticating, and, in rarer cases, the proximal surfaces of some, or all the teeth are abraded. We conclude that this dental abrasion or "surface wear," as Salter styles it, is the result of friction, due to the continual contact of either the neighboring or antagonistic teeth. We are aware that the same effect can be produced by the introduction of certain hard substances into the mouth. Dental abrasion has been discussed by various authors, and is taken into consideration by them when giving a description of normal dentition. But on making a more minute anatomical examination of a large number of mouths we are soon led to the conclusion that certain cases of dental abrasion can no longer be regarded as normal. This peculiar deterioration of the dental organs is worthy of special investigation as to its causes and mode of production, as it is not devoid of interest either to the physician or dental surgeon, as well as to the ethnologist. This fact and the kindness of Prof. B. Eternod, of the Laboratory for Normal Histology and Embryology of the University of Geneva, in placing all the original specimens and casts of his collection pertaining to dental abrasion at my disposal, has prompted me to write on this subject.

The literature on dental abrasion is very extensive, but, unfortunately, most contradictory theories have been advanced, none of which can be considered as a satisfactory definition or plausible explanation of all classes of dental abrasion. This induced me to compare, as conscientiously as possible, the works of other authors. As dental libraries are rarely met with in Europe, it is quite difficult to procure dental literature, but I was successful enough to obtain from the universities of Basle and Geneva, as well as from various private collections enough literary material to enable me to consult some seventy different authors, a summary analysis of which may be found in my dissertation, which appears in the *International Journal of Anatomy and Physiology* of Berlin.

My treatise thus comprises a compilation of the works of other authors as well as my own personal researches and original investigations. I have deemed it necessary to subdivide the paper into the following chapters:

1. Terminology. Dental Abrasion Viewed Anatomically.

2. Microscopic Dental Abrasion. Secondary Dentin.
3. Literature and History.
4. Original Observations.
5. General Discussion of Causes Producing Normal and Abnormal Abrasions.
6. Resulting Modifications; Lesions and Affections.
7. Personal and Authentic Views.
8. Determination of Age in Dental Abrasion.
9. Legal Medicine in Dental Abrasion.
10. Conclusions. Chronological Table of the Authors.

Before entering upon this subject I wish it to be fully understood that I, myself, regard the present treaties simply as a primary contribution to the study of dental abrasion, and also that I wish to resume my investigations in ulterior publications. Unfortunately I was not successful enough to gather sufficient scientific material for determining the causes of chemical abrasion, therefore, I have been entirely confined to the subject of mechanical abrasion.

### Chapter I.

### *Terminology.*

In the general literature on dental abrasion the nomenclature is so inconsistent that the task of collecting literary facts on the subject is quite difficult; for example, different authors—more especially the English—have used the terms "abrasion" and "erosion" synonymously. As I believe this erroneous, I deem it necessary to give their individual definitions.

"*Surface wear*," from the Latin "ursura," implies the removal of material, molecule by molecule, resulting from a rapid, though passive, process of work.

*Abrasion* refers rather to the removal of a substance, slowly or rapidly, but in a more active way.

*Erosion* originally refers to the passive disaggregation of molecules from a uniform mass. But the modern dentist defines dental erosion as a primary anomaly. with further loss of dental tissues, thus deviating from the former signification.

In order to avoid confusion, I shall reserve the terms "surface wear" and "dental abrasion" for normal or abnormal deteriorations of the teeth, apparently resulting from a mechanical friction of contact surfaces. We can now make a distinction between normal abrasion, which comprises all cases of superficial wearing away of the enamel, *i. e.*, *abrasion facets* and abnormal abrasion, encroaching on both enamel and dentine and forming distinct "*abrasion surfaces.*" Abrasion facets are met with constantly after a certain age, while abnormal abrasion surfaces are strictly pathological. Where the nature of the lesion is not precisely stated, I shall simply speak of "*abrasion faces.*" I have deemed this nomenclature indispensable, in order to define the nature of the affection in a clear and precise manner.

Dental abrasion presents itself in various degrees, and the following scale might be advantageously used in designating the degree of affection:

*a. First degree:* Superficial abrasion of the enamel.

*b. Second degree:* Advanced abrasion of the enamel.

*c. Third degree:* Superficial abrasion of the dentine.

*d. Fourth degree:* Abrasion encroaching the pulp-chamber.

*e. Fifth degree:* Abrasion extending to the gingival line.

I have, furthermore, increased the terminology by defining the various forms of abrasion in the following manner:

1. *Planiform abrasion:* When the surface is regular, and spreading out in one direction; this form may be either horizontal or oblique.

2. *Furrow-shaped abrasion:* Occurring on the occluding surfaces of one or more teeth at a time.

3. *Notch-shaped abrasion:* Straight or oblique in individual teeth.

4. *Angular abrasion:* Formed by two planiform abrasions.

5. *Bevel-abrasion:* When a cutting edge is formed by abraded and normal surfaces of the teeth, occurring generally on the anterior surface.

6. *Convex abrasion:* Where the angle is rounded by abrasion.

7. *Cup-shaped abrasion.*

8. *Terrace-shaped abrasion:* Forming a circular salient bordering around the preceding forms of abrasion.

9. *Saddle-shaped abrasion:* Where the abraded surface presents two opposed curves—one concave, the other convex.

According to their anatomical location, we can readily discern abrasion of the masticating and proximal surfaces, as well as abrasion at the gingival point, resulting from mastication, and the use of pipes, cigar-holders, clarionets, bagpipes, etc.

Before entering upon the anatomical description of dental abrasion, I deem it necessary to present the terminology of tooth-surfaces in general. I have, for this purpose, adopted the method prescribed by Tomes. Supposing that the dental parabol be unrolled into a straight line he defines as *anterior surface*, the anterior surface of the incisors, the anterior-exterior surface of the cuspids and bicuspids, and the exterior surface of the molar teeth; he, accordingly, styles the palatal and lingual surfaces as being posterior, whilst here serves the terms "external" and "internal" for the proximal surfaces, according to the way in which they are oriented relative to the mesial plan. I, therefore, recapitulate the proposed terminology in a small synoptic table:

Anterior surface: Vestibulary or facial—labial and jugal.
Posterior surface: Buccal—lingual and palatal.
Exterior surface: Distal, } proximal.
Interior surface: Central or mesial, }

By adopting this clear and simple system of Tomes instead of adhering to the nomenclature usually met with in anatomical and pathological descriptions of the teeth, much confusion is avoided.

### ANATOMICAL CHARACTERS OF DENTAL ABRASION.

When the teeth have first achieved their eruption, they are still covered by the cuticle; their crown are, more or less, armed with cusps, tubercles, prominences, grooves, rugosities, etc. Soon, however, the act of mastication, of friction caused by the lips, cheeks and tongue, and by the teeth upon themselves, encroaches upon this cuticle, effacing completely, in time, the various minor asperities on the surface of the teeth. Later on small, smooth and polished facets are produced, especially at such places where contact of the teeth takes place. These are the forerunners of dental abrasion. This process is constant, and considered normal within certain limits.

But in abnormal and pathological cases this dental destruction progresses beyond the normal limit. It will, therefore, be instructive to give a description of these two categories of abrasion.

A.—*Normal Dental Abrasion.*—This has been the subject of numerous treatises, written by various anatomists; of these, I wish to mention especially Mutilreiteo, Oltramaie and Zuckerkandl. According to Prof. Aebi, Prof. Eternod and Dr. Oltramaie, we must consider the most simple teeth of the human dentition as being formed by reduplication of the primary form, *i. e.*, the union of at least two monocuspid and monoradiculated teeth. According to this theory, the most simple human teeth, such as the incisors and cuspids, were, originally, bicuspid teeth, in which the posterior cusp became nearly or completely atrophied by a phenomenon of resimplification. Likewise the molar teeth are the product of the lateral union of bicuspid teeth. It should be understood, however, that this phenomenon must have taken its origin through serious modifications of the dental follicle. Thus the teeth of each dental arch are composed of two parallel rows of cuspids, placed regularly, one behind the other, in the form of an anterior and a posterior row. We know, furthermore, that the upper teeth work methodically on the lower ones in an alternative manner; thus each tooth occludes with two antagonistic teeth. We are also acquainted with the fact that the superior dental parabol is larger than the inferior. This being the case, we are able to sum up the laws of dental articulation, affrontment and antagonism in the following manner:

1. *Each tooth of one of the jaws corresponds to two teeth of the opposed jaw, the inferior central incisors, perhaps, excepted.*

2. On the whole exposure of the dental parabol, the posterior tubercles of the superior dental arch normally rest on the anterior tubercles of the inferior one.

The anterior cusps are all on the same level, while the posterior cusps are, invariably, on a lower level; this is naturally more pronounced in the cuspid and incisor teeth, especially in the inferior incisors. The distal cusps of the molars are lower than the mesial cusps, and the external angle of all the teeth is lower than the internal angle.

After having studied precisely the general rules concerning the form and relation of the teeth, it will be readily understood that normal abrasion facets occupy constant and perfectly determined positions.

In a general way we can make the following statements:

*First:* Each dental arch has its own characteristic abrasion facets which occupy the posterior range of cusps in the upper jaw, whilst in the lower jaw they are more constantly located on the anterior range of cusps.

*Second:* Each abraded cusp will possess, in principle, one internal and one external facet.

TABLE OF ABRASION FACETS IN A NORMAL HUMAN DENTURE.

| IN THE UPPER JAW. | IN THE LOWER JAW. |
|---|---|
| *Central Incisor:* Two abrasion facets on the masticating surface, that is, on the posterior surface of the anterior cusp; later on they project to the cutting edge, thus abrading the three primary tubercles. | *Central Incisor:* One abrasion facet, destroying the labio-occluding angle and thus forming an oblique planiform surface. |
| *Lateral Incisor:* Two abrasion facets, corresponding with the cusps of the inferior lateral incisor and the inferior cuspid teeth. The affection seldom reaches the cutting border, but in rare cases the dental angle is worn away. | *Lateral Incisor:* Two small planiform oblique abrasion facets formed by the abrasion of the labio-occluding angle; the internal facet occludes with the superior central incisor, and the external facet with the superior lateral incisor. |
| *Cuspid Tooth:* Two abrasion facets in the posterior surface; the internal one being more marked, occludes with the superior cuspid tooth, whilst the dental facet antagonizes with the first bicuspid. | *Cuspid Tooth:* Two abrasion facets on the anterior surface; the internal facet occludes with the superior lateral incisor and the more marked or distal facet with the superior cuspid tooth. |

*Bicuspids:* The abrasion facets are double on these teeth, each of which occlude with two antagonistic teeth.

| | |
|---|---|
| In the *Upper Jaw* the abrasion asserts its influence upon the two cusps, but is more pronounced on the posterior cusp. | In the lower jaw both cusps are affected alike, but the abrasion is generally more marked in the anterior cusps. |

*Molars:* In the molar teeth abrasion begins with a formation of small distinct abrasion facets, resulting from the occlusion of antagonistic cusps, and even from the tubercles in a very perfect dentition.

| | |
|---|---|
| In the *superior molars* the palatal range of cusps is more abraded. | In the *inferior molars* the jugal range of cusps have suffered more distinctly from the effects of abrasion. |

At a certain point in normal dental abrasion the tubercles, and, sometimes, even the cusps disappear, thus allowing far more extensive abrasion-facets.

It is due to their tardy eruption that the third molar teeth rarely present any very marked signs of dental abrasion.

---

In a normal condition the abraded surfaces present an oblique plane, resulting from the mode of articulation. This obliquity can vary in certain limits, according to the individual and the race to which he belongs, and depending on the fact as to whether he bears signs of orthognathism, prognathism, or opistholonhism. There is no doubt that normal dental abrasion varies considerably with the races. These fluctuations in the form and degree of the lesion are all the more comprehensible when it is taken into consideration that the human dentition undoubtedly submits to the influence of ethnical variations with relation to the solidity of the dental tissues. According to the degree of the solidity I have classed the teeth into three principal groups:

1°. *Teeth devoid of much resistance*, having a chalky appearance, with a bluish tint, and always poor in salts.

2°. *Teeth of medium resistance*, whitish and opalescent.

3°. *Teeth of greatest resistance*, indisputably the best, as they possess inorganic salts and organic substances in the proper proportions.

It is a most peculiar and interesting fact that teeth of the latter description are most susceptible to dental abrasion. This is explained by the fact that teeth of greatest resistance are longest intact in the oral cavity. It is also worthy of special notice that teeth with highly-polished normal abrasion facets, and even those with abnormal abrasion-surfaces, are almost exceptionally free from dental caries.

*b). Pathological dental abrasion:* Sooner or later the abrasion of the teeth becomes quite marked, and the real abrasion surfaces take their origin, these are considered as being pathological. In more pronounced cases the abrasion destroys the entire crown (abrasion from the second to the fifth degree). The pathological abrasion surfaces do not necessarily occupy the place that I have assigned to the normal abrasion facets. Their presence is in connection with some special kind of friction, resulting either from a vicious articulation and affrontment of the teeth—the result of abnormal dental arches and defective implantations of the teeth—or from mechanical action of certain foreign substances, such as the end of a pipe, the mouthpiece of a musical instrument, a toothbrush, etc., or from the act of mastication itself.

When abrasion reaches the fourth degree, and the pulp-chamber is encroached upon, one would naturally expect to find the pulp exposed and accompanied by a number of secondary and serious disorders, but, in a majority of cases, this does not happen, as another important phenomenon—the deposition of secondary dentine, creating a defensive barrier of new tissue intervenes, receding in the same measure as the tooth diminishes. This newly-formed dentine is characterized by its density, which is more pronounced than in the normal tissue, also by a variation in color from yellow to dark brown.

When abrasion reaches certain limits, such as to the fifth degree, we often find the pulp-chamber completely obstructed by this newly-formed tissue. Whatever the degree of the lesion may be the abraded surface is invariably smooth, highly polished and without any tendency to dental caries.

In many cases pathological dental abrasion is but the continuation and exaggeration of normal dental abrasion; in these cases the jaws are normal, the dental parabols regular, the teeth well implanted, and the abraded surfaces are mostly planiform, constantly oblique, in their original position, and occupying a normal direction.

In another case, not less frequent, the teeth occlude vertically and directly; the anterior cusps of the inferior teeth do not then occlude with the posterior cusps of the superior teeth as is normally the case, but vertically upon each other. After a certain period their crown adopts a horizontal, truncated appearance, giving the abraded surface generally a planiform, and sometimes even a cupuliform, but rarely a saddle-shaped aspect. In more pronounced cases the process of abrasion becomes so advanced as to reach the neck of the tooth and even destroy the entire crown; in this case the occluding plane maintains a horizontal direction.

In irregular dentition, the articulation of the superior with the inferior teeth is imperfect; normal abrasion facets or abnormal abrasion surfaces can then be produced either separately or simultaneously, thus conferring an undulating, sinuous, tortuous and even angular aspect to the occluding plane.

In abnormal dentition, resulting from the absence of the dental follicle at birth, or destruction of the dental follicle after birth or from the loss of teeth by extraction, caries, etc., in the secondary dentition, abnormal abrasion surfaces are formed instead of normal abrasion facets, due to a modified occlusion of the cusps.

In yet other cases a defective articulation, which results from the abnormal conformation of the dental parabols, causes an imperfect articulation and produces abrasion surfaces varying in form and obliquity.

Abnormal abrasion surfaces may also be formed by an imperfect articulation, which has resulted from a fracture of the jaw.

Lastly, I find in certain cases of either complete or incomplete dentition, that the abrasion does not attack all the teeth on the same level, as in cases where the incisor teeth are abraded down to the gingival margin, while the molar teeth have remained more or less completely intact; this is evidently the result of an abnormal length of the two rami of the inferior maxilla. Frequently the teeth of only one side are abraded, and divers explanations might be given for this condition, such as one-sided mastication, a vicious articulation of the temporo-maxillary joint, etc.

The more work that is required of certain teeth, the quicker they are abraded, *e g.*, if the molars of a superior and inferior secondary dentition are missing, all the work comes on the anterior teeth, resulting naturally in a more marked abrasion of their surfaces.

The abrasions that I have just described refer solely to the masticating surfaces, and are the result of the mutual contact of the two dental arches, we can, however, detect the effects of dental abrasion on the proximal surfaces, as well as on the neck of the teeth.

Proximal dental abrasion exists only in pathological cases; the faces are formed on the proximal surfaces of crowded teeth; their dimensions vary considerably; those of the incisors being generally elongated, the cuspids and bicuspids rounded, and the second bicuspids and molars oval or polygonal.

These proximal abrasion surfaces present the same smooth and polished aspect as those found on the masticating surfaces. They remain within the limit of the first degree, yet must be regarded as abnormal.

The causes of dental abrasion, as found frequently on the necks of the teeth, as well as the singular aspect presented in such cases, generally astonishes the dental surgeon. The lesion almost invariably occupies the incisor teeth, and in time takes the appearance of an angular groove such as might practically be caused by the use of a triangular file.

In marked cases the lesion extends to the dentine, and can even encroach upon the pulp-chamber. Here, as in the preceding forms of this affection, the denuded surface is smooth and polished. In cases where the pulp is reached secondary dentine is constantly forming, as in the previous cases also, thus protecting the vital organ of the tooth from external influences. There is, strangely enough, no tendency to caries in this instance either.

Before closing this anatomical description of normal and pathological dental abrasion, I still wish to mention that there exist abrasions of a professional character, presenting an aspect typical of their causes. Such abrasions can be caused by pipes, cigar and cigarette holders, bagpipes, clarionetts, etc. I also class here cases of abrasion of one or more teeth caused by biting thread or cotton; tailors, seemstresses and bootmakers frequently present such lesions.

